# Efficacy and safety of pentoxifylline for chronic venous leg ulcers: study protocol for a multicenter randomized controlled trial in China (ESPECT study)

**DOI:** 10.1186/s13063-023-07547-y

**Published:** 2023-08-02

**Authors:** Lihong Chen, Yun Gao, Ming Liu, Qiu Li, Chunmao Han, Yue Zhao, Binghui Li, Jun Xu, Yan Dai, Pei Li, Jianli Li, Yuanyuan Li, Xingwu Ran

**Affiliations:** 1 Innovation Center for Wound Repair, Diabetic Foot Care Center, Department of Endocrinology and Metabolism, West China Hospital, Sichuan University, 37 Guo Xue Lane, Chengdu, 610041 China; 2 Department of Peripheral Vascular Disease, Affiliated Hospital of Shandong University of Traditional Chinese Medicine, Jinan, 250014 China; 3 Department of Endocrinology, Shandong Provincial Hospital, Jinan, 250021 China; 4 Department of Burn and Wound Repair, The Second Affiliated Hospital Zhejiang University School of Medicine, Hangzhou, 310009 China; 5 Department of Vascular Surgery, China-Japan Union Hospital of Jilin University, Changchun, 130033 China; 6 Department of Wound Repair, LiYuan Hospital of Tongji Medical College of Huazhong University of Science and Technology, Wuhan, 430077 China; 7 Department of Diabetic Podiatry, Zhu Xianyi Memorial Hospital of Tianjin Medical University, Tianjin, 300070 China; 8 CSPC Ouyi Pharmaceutical Co., Ltd., Shijiazhuang, 052160 China

**Keywords:** Wound and injuries, Venous leg ulcers, Chronic wounds, Pentoxifylline, Wound healing

## Abstract

**Background:**

Venous leg ulcers (VLUs) are the most severe manifestation of chronic venous disease, with long healing time and a high recurrence rate. It imposes a heavy burden on patients, their families, and the health care system. Chronic inflammation triggered by sustained venous hypertension is now recognized as the hallmark of chronic venous disease. The anti-inflammatory effect of pentoxifylline may offer a promising avenue to treat VLUs. However, current evidence of pentoxifylline for VLUs is relatively small and of low quality. The aim of this study is to evaluate the efficacy and safety of pentoxifylline for VLUs in the Chinese population.

**Methods:**

This is a randomized, double-blinded, double-dummy, multi-center, placebo-controlled clinical trial. A total of 240 patients will be randomized to receive pentoxifylline (400 mg, twice daily) or placebo for 24 weeks. All participants will receive diosmin treatment and standard care of VLUs and other comorbidities. The primary outcome is the difference in the wound healing rate within 12 weeks between pentoxifylline and placebo. Secondary outcomes include (1) percent wound size changes at 12 weeks, (2) the levels of TNF-α and IL-6, (3) venous clinical severity score and chronic venous insufficiency quality of life score, and (4) ulcer recurrence within 24 weeks.

**Discussion:**

This study would evaluate the efficacy and safety of pentoxifylline for VLUs in the Chinese population. If confirmed, it wound offer another effective and safe therapeutic option for treatment of VLUs.

**Trial registration:**

The trial was registered at the Chinese Clinical Trial Registry (No. ChiCTR-2100053053). Registered on 10 November, 2021, https://www.chictr.org.cn/showproj.aspx?proj=137010

## Background

Chronic venous disease (CVD) is a common pathology of the circulation system and is characterized by symptoms and signs as a result of structural or functional abnormalities of the veins. The most common symptoms and signs include pain, tightness, heaviness, feeling of swelling, reticular and varicose veins, edema, skin changes such as pigmentation, or ulceration [1]. CVD is often overlooked by healthcare providers because of an under-appreciation of the magnitude and impact of the problem, as well as incomplete recognition of the manifestations. In fact, CVD is a very common problem. A global survey suggested that the worldwide prevalence of CVD is 83.6%, with 63.9% of the subjects ranging from C1 to C6 [2]. In China, the prevalence of CVD is 8.89%, that is, there are nearly 100 million patients. The annual incidence rate is 0.5% to 3.0% [3].

Venous leg ulcers (VLUs) are defined as ulcers of the leg or foot that occur in the presence of venous disease. VLUs are the most severe manifestation of CVD. VLUs represent 80% of all leg ulcers [4]. The prevalence of VLUs is 1.5% in the general population in China [3], with a higher prevalence in the elderly [4]. Furthermore, VLUs are characterized by slow healing time and a high recurrence rate [5]. With the advent of global aging, more populations will be affected by VLUs. VLUs impose a heavy burden on patients’ physical and mental health and substantially reduce their quality of life. In addition, VLUs impose a heavy socioeconomic burden on patients, their families, and the health care system. Therefore, it is imperative to take efforts to improve the management of VLUs.

The main pathophysiology of VLUs is venous reflux or obstruction, venous hypertension, and chronic inflammation [6]. Conservative management is often the initial choice to reduce symptoms and protect against the progression of CVD. Given the severity and anatomic disorders of CVD, surgical and interventional methods are often reserved for unsatisfactory responses to conservative measures. Compression therapy is the mainstay of CVD [7]. Compression therapy can significantly improve symptoms and signs such as pain, swelling, skin pigmentation, activity, and well-being. For patients with ulcers, the benefits of long-term compression therapy have been repeatedly demonstrated in randomized trials. A healing rate as high as 97% can be achieved in those who are compliant with therapy [8].

In addition, pharmacological treatment could be an option. Venoactive drugs include flavonoids (diosmin), aescin, and coumarins. The main principle of these drugs is to improve venous tone, capillary permeability, lymphatic drainage, and muscle pump function. These drugs could provide relief of pain and swelling or accelerate venous ulcer healing. The nonvenoactive drug sulodexide has also been used to treat VLUs effectively [9]. In recent years, it has been increasingly recognized that chronic inflammation, possibly triggered by sustained venous hypertension, is the principal basis that potentiates the disease progression of CVD. Chronic inflammation of the venous vessel wall could lead to degradation of venous integrity and function, resulting in diminished venous return, fluid accumulation, tissue fibrosis, atrophy, and ulceration in severe cases [10].

Pentoxifylline is used as a drug to treat peripheral artery disease and has been used in the treatment of VLUs. It could inhibit the synthesis of inflammatory mediators, decrease cytokine release, and suppress leukocyte function [11]. The anti-inflammatory effect of pentoxifylline may offer a promising avenue to treat VLUs. In fact, there have been several trials suggesting that pentoxifylline could improve the healing rates of VLUs. In a meta-analysis of 13 randomized clinical trials, pentoxifylline significantly improved the ulcer healing rate and time to healing of VLUs [12].

In addition, other possible mechanisms of pentoxifylline might improve wound healing [13]. Briefly, pentoxifylline could increase cAMP levels in the smooth muscle of vessels, increase erythrocyte flexibility, and then improve microcirculation and oxygen delivery [14]. The fibrinolytic property might degrade microcirculation fibrin, improve skin and subcutaneous nutrition, and then improve wound healing [15].

However, the evidence that currently recommends the use of pentoxifylline in VLUs is relatively weak. Furthermore, none of the trials were carried out in China. Thus, it is imperative to investigate the efficacy and safety of pentoxifylline for VLUs in the Chinese population. Therefore, we designed a multi-center, randomized, double-blinded, placebo-controlled clinical trial to evaluate the efficacy and safety of pentoxifylline in the Chinese population for the treatment of VLUs.

## Methods and design

### Objective

The main aim of this study is to investigate the efficacy and safety of pentoxifylline for VLUs in the Chinese population.

### Study design

**Fig. 1:**
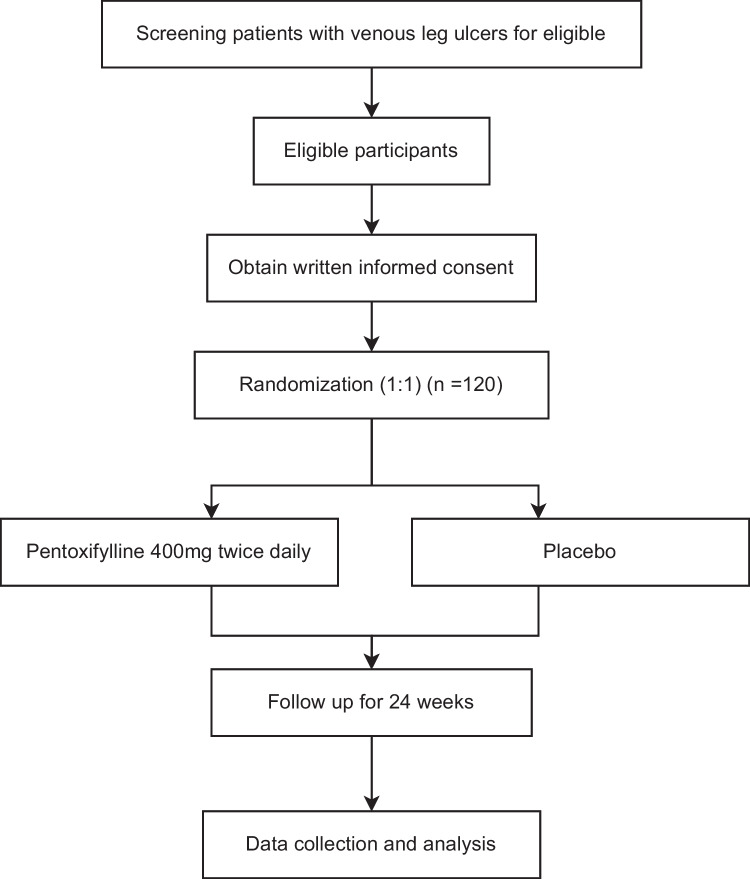
Flow chart of the pentoxifylline clinical trial

This is a multicenter, randomized, double-blinded, double-dummy, placebo-controlled clinical trial. The study was registered at the Chinese Clinical Trial Registry (No. ChiCTR-2100053053). Eligible participants will be randomly allocated to either pentoxifylline or placebo at a ratio of 1:1. The flow chart of this trial is briefly shown in Fig. 1. The protocol was designed according to the Standard Protocol Items: Recommendations for Interventional Trials (SPIRIT) 2013 Statement [16].

### Study population

Recruitment of patients with VLUs will be conducted at thirteen hospitals in China (Table 1). All patients seeking treatment of VLUs in these hospitals will be screened according to the inclusion and exclusion criteria. Once written consent is obtained and the inclusion criteria is confirmed, participants will be enrolled in this trial. This trial will begin in August 2022 and will continue until October 2024.

**Table 1:** The hospitals participating in this study

Number	Participating hospitals
1	West China hospital, Sichuan University
2	Sichuan Provincial People’s Hospital
3	Shanxi Bethune Hospital
4	Liyuan Hospital of Tongji Medical College of Huazhong University of Science & Technology
5	Zhu Xianyi Memorial Hospital of Tianjin Medical University
6	Tianjin First Central Hospital
7	China-Japan Union Hospital of Jilin University
8	Shandong Provincial Hospital
9	The Affiliated Hospital of Shandong University of Traditional Chinese Medicine
10	The Affiliated Hospital of Qingdao University
11	The Second Affiliated Hospital Zhejiang University School of Medicine
12	Jiangsu Province Hospital
13	Nanfang Hospital, Southern Medical University

### Diagnostic criteria of VLUs

VLUs are defined as ulcers of the leg or foot that occur in the presence of venous disease. CEAP classification is a widely accepted tool to diagnose CVD [17]. The name CEAP classification stands for Clinical (C), Etiological (E), Anatomical (A), and Pathophysiological (P). VLUs is the most advanced form of CVD [18]. In this trial, CEAP was used to assist the diagnosis of VLUs.

### Inclusion criteria

To be eligible to participate in this study, a subject must meet all of the following criteria:

1. Age ≥ 18 years
2. Meeting the criteria of VLUs (patients with CVD and complicated with ulcers (level C6 of CEAP classification). The surface area of the VLU should be within the range of 1 cm^2^ and 20 cm^2^. If there are more than one ulcer in a subject, the ulcer with the largest area will be chosen as the index ulcer. The distance between the index ulcer and other ulcers should be more than 2 cm.
3. Consciousness, without aphasia, able to eat drugs
4. Ankle brachial index ≥ 0.7
5. Written informed consent by participants or surrogate

### Exclusion criteria

A potential subject who meets any of the following criteria will be excluded from this trial:

1. Ulcers of other etiologies, such as sickle cell anemia, necrobiosis lipoidica, pyoderma gangrenosum, lower extremity artery disease, and vasculitis
2. Known malignancies, or immune deficiency disease
3. Severe infection
4. Known pregnancy or lactation, or patients who anticipated becoming pregnant
5. Allergy to pentoxifylline or methylxanthines
6. Gangrene of ulcers
7. Lower extremity edema caused by cardiac, hepatic, or renal disease
8. Known severe disorder of heart, liver, kidney or hematopoietic system (ALT more than 2.5 times upper limit of normal, AST more than 2.5 times upper limit of normal, or creatine more than 1.5 times upper limit of normal)
9. Known acute cardiac infarction, severe coronary artery disease, cerebral arteriosclerosis, hypertension, or severe arrhythmia
10. Known cerebral hemorrhage, subarachnoid hemorrhage, retinal hemorrhage, hemorrhagic stroke, bleeding tendency (for example, long-term use of anticoagulant drugs), or history of recent bleeding

### Withdrawal of individual subjects

Subjects can leave the study at any time for any reason. The trial should be suspended in a special subject in the following conditions: (1) serious adverse events relevant or not relevant to pentoxifylline occur, (2) suffer from acute life-threatening disease, (3) death, and (4) violate the trial protocol, which influenced the evaluation of the endpoint.

### Informed consent

Written informed consent will be obtained from all participants at each participating center or their legal representative if they are unable to provide consent. On the consent form, participants will be asked whether they agree to the use of their data if they opt out of the trial. Participants will also be asked for permission for the research team to share relevant data with people from the universities taking part in the research or from regulatory authorities, where relevant. Clinical trial liability insurance will be purchased for participants during the trial treatment period. After the trial ended, trial participants will still have access to their attending doctors. Because there is no anticipated harm, no compensation for trial participation will be provided. Informed consent materials of Chinese version (not in English) are available, on request, from the corresponding author.

### Randomization and allocation concealment

All eligible participants will be randomly assigned (1:1) to either the pentoxifylline group or the placebo group. The random sequence will be generated by an interactive response technology (IRT) system of a third-party contract research organization (CRO). Drug numbers are marked by personnel independent of the clinical trial. After a subject passes the screening procedure, the IRT will assign the subject a random number that is used to associate the patient with the drug number. This unique drug number will be displayed on the packaging of the study drug assigned to the patient. The random number is associated with the drug and the treatment group by the IRT system. In addition, the IRT could effectively guarantee the implementation of blinding. All participants, primary investigators, healthcare providers, laboratory staff, and statisticians will be blinded until the end of the research. If serious adverse effect occurs, unblinding would be permitted.

### Interventions

All patients will receive standard care of VLUs including compression therapy, sclerotherapy, and endovenous and surgical management. Other standard management such as treatment of infection, debridement, local wound care, anti-inflammatory analgesic drugs, dressings, and metabolic management will be prescribed as appropriate. Current guidelines were followed as per the attending clinician’s judgment [3, 5].

After randomization, participants will be given either pentoxifylline 400 mg twice daily or matched placebo (simulation agent of pentoxifylline) for 24 weeks. Because the dosage of pentoxifylline was 400 to 800 mg per day on the label of pentoxifylline in China, the dose in the trial was 400 mg twice a day. The micronized purified flavonoid fraction (90% diosmin and 10% other flavonoids) is currently one of the most widely accepted effective drugs for CVD and is also recommended by guidelines [17]. Thus, all patients in both groups will be dosed 450 mg diosmin twice daily for 24 weeks. Participants are prohibited from using fibrinolytic drug sulodexide and other venoactive drugs, such as aescin (horse chestnut extracts), coumarins, and low molecular weight heparin. All participants will be required to return the drug tablet to monitor the adherence. There will be no special criteria for discontinuing or modifying allocated interventions.

### Outcomes

#### Primary outcome

Wound healing rate within 12 weeks, expressed as the proportion of participants with complete wound healing of the index ulcer within 12 weeks. Complete wound healing is defined as full epithelialization of the wound without scarring [19].

#### Secondary outcomes

1. The wound healing rate of other periods (4, 8, 16, and 24 weeks)
2. Percent of wound size changes at 12 weeks
3. The levels of TNF-α and IL-6 at 4, 12, and 24 weeks
4. Venous clinical severity score (VCSS) at 12 and 24 weeks
5. Chronic venous insufficiency quality of life score (CIVIQ) at 12 and 24 weeks
6. Ulcer recurrence within 24 weeks for subjects whose index ulcer heals by conclusion of 12-week visit

### Follow-up

**Fig. 2:**
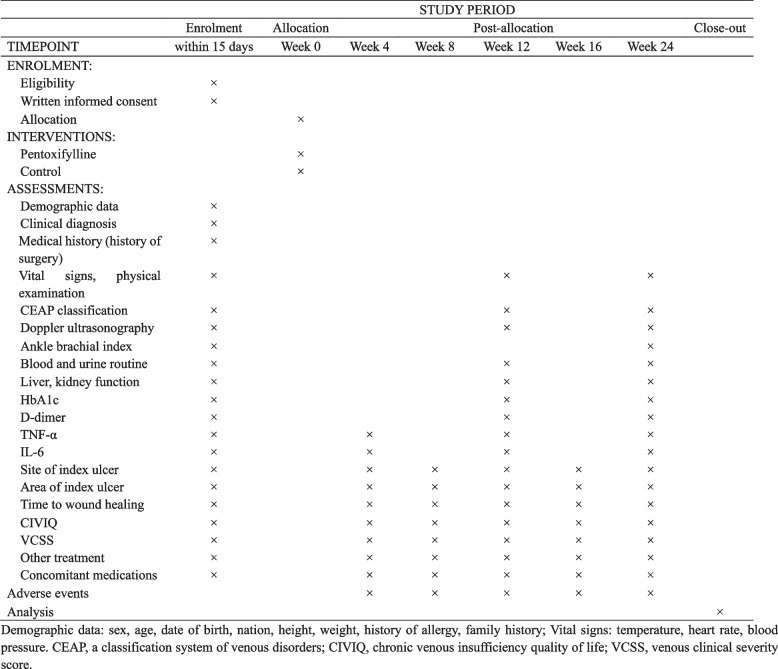
Schedule of enrolment, interventions, and assessments

The total follow-up period is 24 weeks post randomization. The items to be measured and the time window of data collection are shown in Fig. 2.

### Adverse events

All adverse events related to or unrelated to this study, reported spontaneously by the subject or observed by the research investigator, will be recorded. The common terminology criteria for adverse events v5.0 will be used as the criteria of classification of adverse events. Serious adverse events will be reported to the research center expert committee and ethics committee within 24 h. Any adverse event that occurs in the clinical trial will be treated uniformly according to the severity of the adverse event. Follow-up of adverse events will be followed through 12 months after initiation of treatment.

### Sample size

The aim of this study is to investigate the effect of pentoxifylline on the wound healing rate of VLUs within 12 weeks. According to a recent meta-analysis, the wound healing rates of the pentoxifylline treatment and the control group were 62.7% and 40.6%, respectively [12]. A total of 190 participants will be required at the 5% significance level and 80% power. Assuming a drop-out rate of 20%, 240 participants will be included in this trial.

### Data collection and management

Before the start of the study, all primary investigators at different centers will discuss the research protocol face-to-face or online. Meetings will be held to train researchers who will participate in the trial. The principal investigator of the research unit is responsible for the implementation and management of this clinical trial. Other researchers are responsible for clinical diagnosis/treatment, informed consent, screening, clinical sample collection, follow-up, data entry, etc. The pharmacy administrator of the research unit is responsible for the management of clinical trial drugs. A researcher will collect data through a prespecified case report form (CRF). The data should be accurate, complete, timely and reliable. A clinical trial associate will verify whether the data filled in the CRF are complete and accurate. EDC system is used to input data in CRF, which is proofread by data administrator. Interim analysis will not be adopted. The sponsor shall provide clinical inspectors for monitoring (1–2 times per month). An independent third-party company is responsible for the compilation of experimental drugs, data statistics, etc. All personal information of participants collected will be remained strictly confidential. The primary investigator has the access to the final trial dataset.

### Data analysis

An independent statistical analysis plan will be formulated. All analyses will be conducted in the full analysis set (FAS) population, defined as all patients randomized to an intervention group having at least one efficacy treatment after randomization. The outcomes will also be assessed in the per-protocol set (PPS). The results from FAS and PPS will be compared. Safety set will be used to evaluate the safety of experimental drug. The compliance of treatment and treatment-related withdrawals will be evaluated by arm. Participants’ characteristics and clinical measures will be described as the mean ± standard, median (interquartile range), or frequencies and percentages as appropriate. All statistical analyses will be performed using SAS, V.9.4. All statistical tests are bilateral tests. A *p* value ≤ 0.05 is considered statistically significant.

### Analysis of efficacy

The primary analysis will aim to compare the wound healing rate within 12 weeks between the pentoxifylline group and the control group by chi-squared test or Fisher’s exact test. The result will be expressed as risk ratio and 95% confidence interval. The wound healing rate at 4, 8, 16, and 24 weeks will be assessed by time-event analysis. Kaplan–Meier plot, Log-rank test, and Cox regression will also be used to analysis the time-to-event outcome, with the result of hazard ratio and 95% confidence interval. The covariates that should be adjusted in the Cox regression analysis include gender, age, duration of disease, BMI, pre-treatment ulcer area, compression therapy, and endovenous surgery. Last observation carried forward will be used to fill up the missing values if some primary outcomes may be missing.

The secondary analysis will compare the VCSS score, CIVIQ score, percent of wound size changes, and ulcer recurrence within 24 weeks by *t* test, Wilcoxon rank test, chi-squared test, or Fisher’s exact test as appropriate.

### Analysis of safety

The safety data will be described as frequencies and percentages and analyzed by chi-squared statistics and Fisher’s exact test as appropriate.

### Patient and public involvement

Patients and the general public were not involved in the development of the research question, outcome measures, or protocol design.

### Protocol amendments

Before the change of the clinical trial protocol, the principal investigator of the group leader unit should conduct a comprehensive and in-depth research and evaluation on the safety risks of the subjects and the scientific nature of the change of trial protocol, so as to judge the nature of the change scientifically and reasonably, and distinguish between substantive and non-substantive changes. For substantive changes, further clarification is needed as to whether they would significantly increase safety risk for clinical trial participants. When the protocol is changed during the drug clinical trial, on the basis of full risk assessment by the principal investigator, the relevant regulations and requirements of ethical review shall be strictly observed. If necessary, the informed consent and other relevant documents should be updated and reported to the ethics Committee for review, and the updated relevant documents should be sent to the principal investigator and the ethics committee of the sub-center.

## Discussion

Pentoxifylline is a readily available, safe, and inexpensive medication that was approved for the treatment of peripheral vascular disease. There were a few of clinical studies supporting a role for pentoxifylline in VLUs. However, the current studies enrolled a small number of subjects and were of short duration. This trial might provide more definite evidence that whether pentoxifylline could be an effective and safe therapeutic option for treatment of VLUs.

VLUs is the most common etiology of lower extremity ulcers followed by peripheral artery disease [4, 20]. It is postulated that the coexistence of both venous and arterial diseases accounted for 26% of patients with lower extremity ulceration [21]. This kind of mixed leg ulcer pose a particular challenge in clinical practice, because they are recalcitrant due to the different underlying pathologies [22]. However, due to the multiple effects of pentoxifylline on both venous and arterial disease, the results of this study might introduce a new choice for mixed arterial-venous ulcers.

Currently, the overall prognosis of VLUs is poor. Although compression therapy is the mainstay of CVD, supplementary therapy might have additional benefit. Combination therapy of venoactive drugs and other medications with different mechanism are usually considered in clinical practice. However, there was little evidence that support combination therapy for VLUs. Because the micronized purified flavonoid fraction (90% diosmin and 10% other flavonoids) is currently strongly recommended in the guidelines of CVD with moderate evidence [1], all subjects will be prescribed diosmin in this trial. The results of this study might provide evidence that whether the combination therapy of diosmin and pentoxifylline could have better effect than diosmin single drug treatment.

In this study, standard of care of VLUs is determined by the treating physicians and not protocolized. This is similar with real world practice. Thus, it is assumed that the results could have good generalization to the general Chinese population.

There are some limitations in this trial. The study will only be performed in the Chinese population. It is unclear whether the results of this trial could be generalized to people in other areas. Second, ulcer recurrence will be followed up for only 24 weeks. The time period is relatively short to observe ulcer recurrence. Third, we will use diosmin as a background drug in this study. Although it is currently assumed effective, it was not widely used in the worldwide. This might have a limitation to external validity.

### Trial status

This protocol is the first version, approved on August 23, 2021. Recruitment of participants will begin on August 2022. The study is scheduled for completion in October 2024. The findings of this study will be published by the investigators in relevant scientific peer-reviewed journals.

## Data Availability

Not applicable.

## Abbreviations

CEAP: A classification system of venous disorders
CIVIQ: Chronic venous insufficiency quality of life
CRF: Case report form
CVD: Chronic venous disease
FAS: Full analysis set
PPS: Per-protocol set
VCSS: Venous clinical severity score
VLU: Venous leg ulcer
